# Scientific and technological contributions of Latin America and Caribbean countries to the Zika virus outbreak

**DOI:** 10.1186/s12889-019-6842-x

**Published:** 2019-05-09

**Authors:** Alice Machado-Silva, Camila Guindalini, Fernanda Lopes Fonseca, Marcus Vinicius Pereira-Silva, Bruna de Paula Fonseca

**Affiliations:** 1 Centro de Desenvolvimento Tecnológico em Saúde (CDTS), Fiocruz, Rio de Janeiro, Brazil; 2 Casa de Oswaldo Cruz, Fiocruz, Rio de Janeiro, Brazil; 3 Instituto René Rachou, Fiocruz, Minas Gerais, Brazil; 4 Observatório em Ciência, Tecnologia e Inovação em Saúde da Fiocruz, Rio de Janeiro, Brazil

**Keywords:** Zika, Latin America, S&T, Epidemic, Patents, Publications, Co-authorship networks

## Abstract

**Background:**

The recent Zika virus (ZIKAV) epidemics disclosed a major public health threat and a scientific and technological (S&T) challenge. The lessons learned from the S&T response of Latin America and the Caribbean (LAC) countries are critical to inform further research and guide scientific investments. The present study aimed to assess how new S&T knowledge produced and disseminated regionally can contribute to address global health challenges.

**Methods:**

Scientometric and social network analysis methods were used to assess the LAC scientific contribution and potential technological development on ZIKAV up to December 2017. ZIKAV-related publications were retrieved from the Web of Science, Scopus, and PubMed databases. Regionally published articles were obtained from SciELO (Scientific Electronic Library Online) and LILACS (Literature in the Health Sciences in Latin America and the Caribbean) databases. Patent registries were retrieved using Orbit Intelligence and Derwent Innovation. Records from each database were individually downloaded, integrated, standardized and analyzed.

**Results:**

We retrieved 5421 ZIKAV-related publications, revealing a sharp increase from 2015 onwards. LAC countries accounted for 20% of all publications and Brazil was among the top three most central countries in the global network for ZIKAV research. A total of 274 patent families backed up by experimental evidence were retrieved. Only 5% were filed by LAC assignees, all of them based in Brazil. The largest contribution of LAC research was on the clinical manifestations of the ZIKAV infection, along with vector control, which was also the main focus of patents.

**Conclusions:**

Our analysis offered a comprehensive overview of ZIKAV’s research and development and showed that (i) LAC countries had a key role in generating and disseminating scientific knowledge on ZIKAV; (ii) LAC countries have expressively contributed to research on ZIKAV clinical manifestations; (iii) the Brazilian scientific community was potentially very effective in knowledge sharing and diffusion in the ZIKAV research network; (iv) Brazil was the single LAC country filing patents, mostly represented by independent inventors and low-tech patents. The paper advocates the need for a continued interdisciplinary approach to improve LAC countries ability to prevent, prepare for and control future outbreaks.

**Electronic supplementary material:**

The online version of this article (10.1186/s12889-019-6842-x) contains supplementary material, which is available to authorized users.

## Background

The Zika virus (ZIKAV) was considered harmless until the Guillain-Barré syndrome and other autoimmune complications were shown to be associated with the infection during the French Polynesia outbreak in 2013–2014 [1]. The 2015 Latin America and the Caribbean (LAC) outbreak increased its scientific and public health interest worldwide [2]. Over one million people were infected in the region, with a shocking number of microcephaly cases in fetuses and infants [3, 4]. Given the epidemic extension and the identification of ZIKAV as the causal link of congenital neurodevelopmental defects, the World Health Organization (WHO) recognized the ZIKAV infection as a public health emergency of international concern (PHEIC) [5]. In 2017, it was estimated that the ZIKAV disease was responsible for 2.24 thousand disability-adjusted life years (DALY) globally [6].

Despite significant advances, ZIKAV remains an enduring scientific and technological (S&T) health challenge. So far, the physiopathology and broad spectrum of clinical manifestations have not been completely understood. There is a lack of reliable diagnostic assays, and no vaccine or specific anti-viral treatment available. Though these technological advances are expected shortly [7], ZIKAV is likely to have significant and long-lasting social and economic impacts across LAC [8].

Previous bibliometric studies have shown that scientific publications on ZIKAV have greatly increased in the last few years [9–12]. However, not much is known about the potential S&T contributions of LAC countries. It has been suggested that expanding the science, technology and innovation base in developing countries, as well as their insertion in research networks, would improve their response and preparedness to emerging health threats of global concern [13]. An assessment of how LAC countries responded to the scientific challenge imposed by the ZIKAV outbreak can provide useful information to the global health community, and guide the prioritization of research and financial investments.

In this paper, ZIKAV-related publications and patents were reviewed to examine: i) LAC’s contribution to the worldwide scientific knowledge production and potential technological development; ii) research areas in which LAC countries mostly contributed; iii) role of LAC countries in the research network; iv) LAC patenting profile. This information is expected to contribute to the discussion of the role of S&T knowledge produced and disseminated regionally to address global health challenges.

## Methods

### Scientific publication analyses

#### Data collection and search strategy

ZIKAV-related scientific publications (up to December 2017) were retrieved from three international databases: Web of Science (WoS), Scopus and PubMed. The query was directed to the title, abstract and keywords using the search terms “zika OR zikav OR zikv”. Only articles, reviews, editorials, letters and notes were included in the analysis. Replies, errata, proceedings papers, meeting abstracts, books, and comments on existing papers were excluded.

To account for regional or local publications, data from SciELO (Scientific Electronic Library Online) and LILACS (Literature in the Health Sciences in Latin America and the Caribbean) databases were collected using the same search strategy. SciELO, originally from Brazil, indexes 1456 journals, 86% of them from LAC countries. LILACS covers 918 medicine and health sciences journals from the LAC region. LILACS is maintained by the Latin American and Caribbean Center on Health Sciences Information (BIREME) of the Pan American Health Organization.

#### Cleaning and standardization of data

Records from each database were downloaded separately and then integrated. Duplicates were removed and the data harmonized into a single dataset using the “data fusion” tool of the VantagePoint software (Search Technology Inc.). The procedure was done separately for Scopus, PubMed and WoS records (hereinafter Global dataset), and then for SciELO and LILACS databases (hereinafter Regional dataset). The Regional dataset was cross-checked with the Global dataset to assess the international coverage of regionally indexed ZIKAV publications and control for potential biases in the assessment of LAC contributions. The Global dataset used is available as Additional file 1.

First authors or co-authors’ affiliation data available in the Global dataset were used to assign publications to their respective countries. Names of institutions were standardized to ensure the correct acknowledgment of their scientific production using the “list clean up” function of the VantagePoint software. The organization-enhanced list produced by the WoS was used as a reference to group departments and institutions.

#### Thematic mapping and clustering

A combined approach of mapping and clustering was used to provide an overview of research themes present in the Global dataset. Term maps were constructed using the visualization of similarities mapping technique available on the VOSviewer software [14], using the “association strength” measure as proposed by Van Eck and Waltman [15]. In summary, two terms are deemed to be strongly related if they frequently co-occur in publications. In the map, each term is represented by a circle, and the closer they are positioned, the more related they are. The diameter and label size of each circle indicate the number of occurrences of the corresponding term in the title or abstract of publications. A weighted and parameterized variant of modularity-based clustering is used by the software to identify clusters of related terms [16].

#### Co-authorship network analysis

Authors’ affiliation data available in the Global dataset was used to assess country collaboration dynamics through the analysis of co-authorship networks. In these networks, nodes represent countries, and two or more countries were connected if their members shared authorship of one or more papers. As co-authorship requires reciprocal cooperation among participants, all connections have been considered as non-directional. Visualization of network graphs and statistical analyses were produced with the open-source software Gephi [17].

Countries that had prominent roles in the network were identified by their betweenness centrality, which reflects the extent a node acts as a “bridge” between other nodes [18]. Central countries usually have a broker position as they connect many other nodes, and thus have more access and control over resources, leading knowledge exchange and preventing others from isolation.

### Patent analyses

#### Data collection and search strategy

Patent searches were carried out using Orbit Intelligence (Questel) and Derwent Innovation (Clarivate) commercial databases, between March and August 2018. The search strategy focused on patent applications that had the earliest priority date up to 12/31/2017 filed anywhere in the world. The search included all documents containing “zika OR zikav OR zikv” in their title, abstract or claims. Results from Derwent Innovation were integrated into Orbit Intelligence. An additional file provides the raw dataset retrieved (see Additional file 2).

#### Grouping patent families

The patent documents were grouped into FamPat patent families, which include documents that are believed to cover the same invention. This grouping is automatically made by Orbit Intelligence and cover, for instance, different stages of an application in a given country or related applications that are filed in different countries.

#### Cleaning and standardization of data

Patent families were manually reviewed to exclude inventions outside the search scope or not showing experimental evidence related to ZIKAV. Patent assignee names were standardized using Orbit Intelligence grouping functionality. Alternative spellings and subsidiaries were grouped under a single name. Further manual cleaning was conducted to include the research institution’s name when other bodies (i.e., the university’s funding agency, the board of regents or technology transfer office) appeared as the patent assignee.

#### Identification of R&D country

Inventor and assignee addresses were used to infer where research and development (R&D) took place. If no information on address was available, the earliest priority country was considered (i.e., the country where the first patent application from the respective family was filed).

### Markets of interest

Orbit Intelligence’s analysis module lists all countries where protection for the invention was sought (based on country of filing). Only countries where patents were still alive, either granted or pending, were considered for this analysis.

### Assignee classification

The assignees were manually classified as “academy” (universities, research institutes, and other not-for-profit entities), “corporate” (companies) or “independent inventors” (assignees with no institutional affiliation). Assignees were accounted for every time they were nominated, even if they appeared in a previous patent family.

### Classification of technologies

Technologies were classified using specific international patent codes (CPC – Cooperative Patent Classification and IPC- International Patent Classification) and keywords in the patents’ title. The classification was verified through the review of the full patent description.

## Results

### LAC countries have greatly contributed to knowledge production on ZIKAV

To assess the contribution of LAC to the worldwide knowledge production on ZIKAV, searches for scientific publications were carried out in international databases: 4232 documents were retrieved from Scopus, 3378 from PubMed and 4001 from the WoS. After data integration, standardization, and treatment, 5421 unique documents were included in the analysis (Global dataset). Scopus and WoS had the highest number of unique records, with approximately 16% of all publications each (see Additional file 3, left panel). Overall, 50% of all publications retrieved were indexed in all three databases.

To evaluate the international coverage of regionally indexed ZIKAV-related publications and control for potential biases in the assessment of LAC contributions, searches were also carried out in regional LAC databases. The search in the regional databases retrieved 193 records from SciELO and 157 from LILACS. After treatment and processing, 237 unique publications were identified (Regional dataset). There was an overlap of 48% between the two databases; 70% of the publications were also indexed in the international databases (see Additional file 3, right panel).

**Fig. 1:**
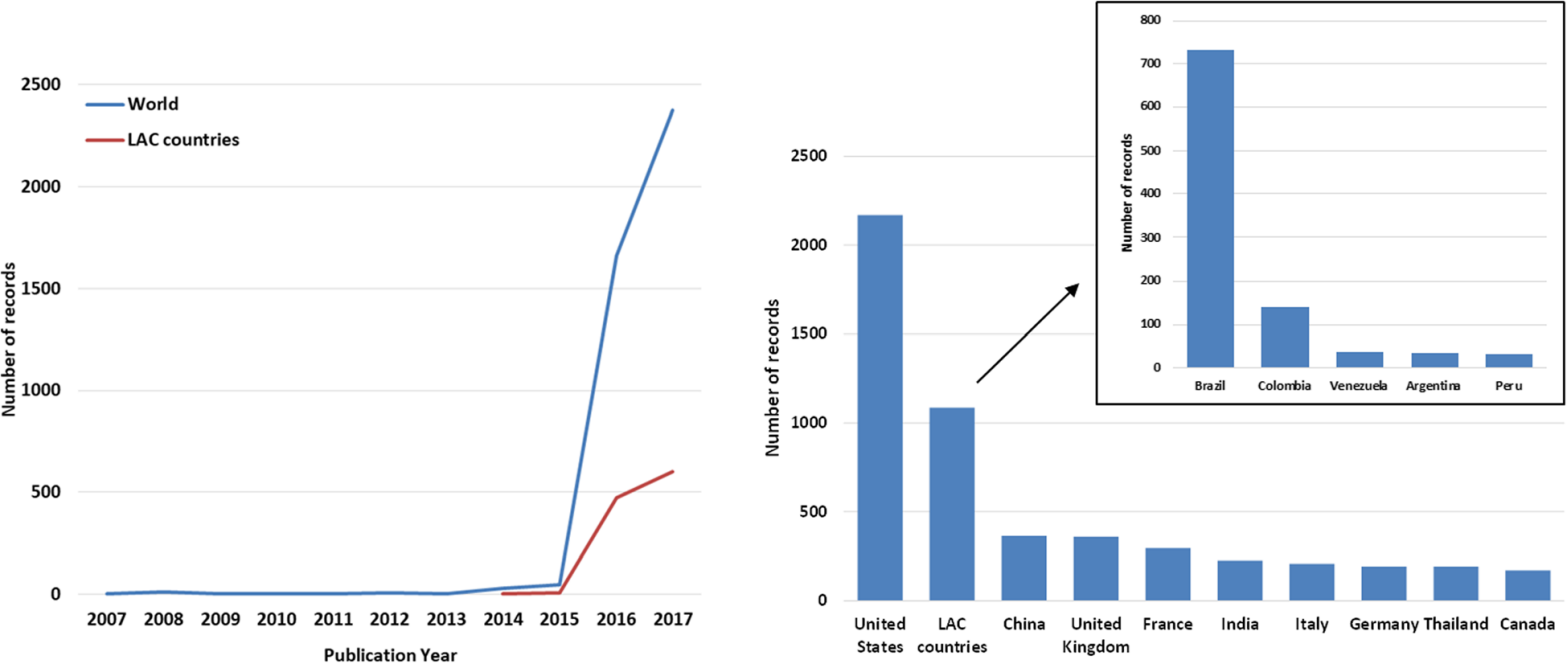
ZIKAV scientific publications indexed in international databases (2007–2017). Left panel: annual number of published articles on ZIKAV; Right panel: top ten most productive countries/regions

Our data showed a sharp increase in ZIKAV publications over the last years, particularly from 2015 onwards, both worldwide and in LAC countries, (Fig. 1, left panel). Authors from the USA (*n* = 2169), LAC countries (*n* = 1086) and China (*n* = 365) were the most frequent in the period reviewed (Fig. 1, right panel). Brazil was involved in 67% of all LAC publications (*n* = 729), accounting for 13% of the world’s scientific publications on ZIKAV, followed by Colombia (*n* = 140), Venezuela (n = 36) and Argentina (*n* = 35) (Fig. 1, right panel).

The top 10 institutions according to the number of published articles are presented in Table 1. Fundação Oswaldo Cruz (Fiocruz), from Brazil, was the most frequent contributor with a total of 243 publications, followed by the University of California, US Centers for Disease Control and Prevention (CDC), Universidade de São Paulo and Harvard University with 222, 220, 149, 147 published articles, respectively (Table 1).

**Table 1:** Top ten institutions according to the number of published articles on ZIKAV (2007–2017)

Rank	Institution	Country	Number of records
1	Fundação Oswaldo Cruz	Brazil	243
2	University of California	USA	222
3	Centers for Disease Control and Prevention (CDC)	USA	220
4	Universidade de São Paulo	Brazil	149
5	Harvard University	USA	147
6	Institut Pasteur	France	145
7	Hainan Medical University	China	128
8	Johns Hopkins University	USA	110
9	University of Texas System	USA	109
10	Universidade Federal do Rio de Janeiro	Brazil	97

### LAC countries have expressively contributed to research on ZIKAV clinical manifestations

**Fig. 2:**
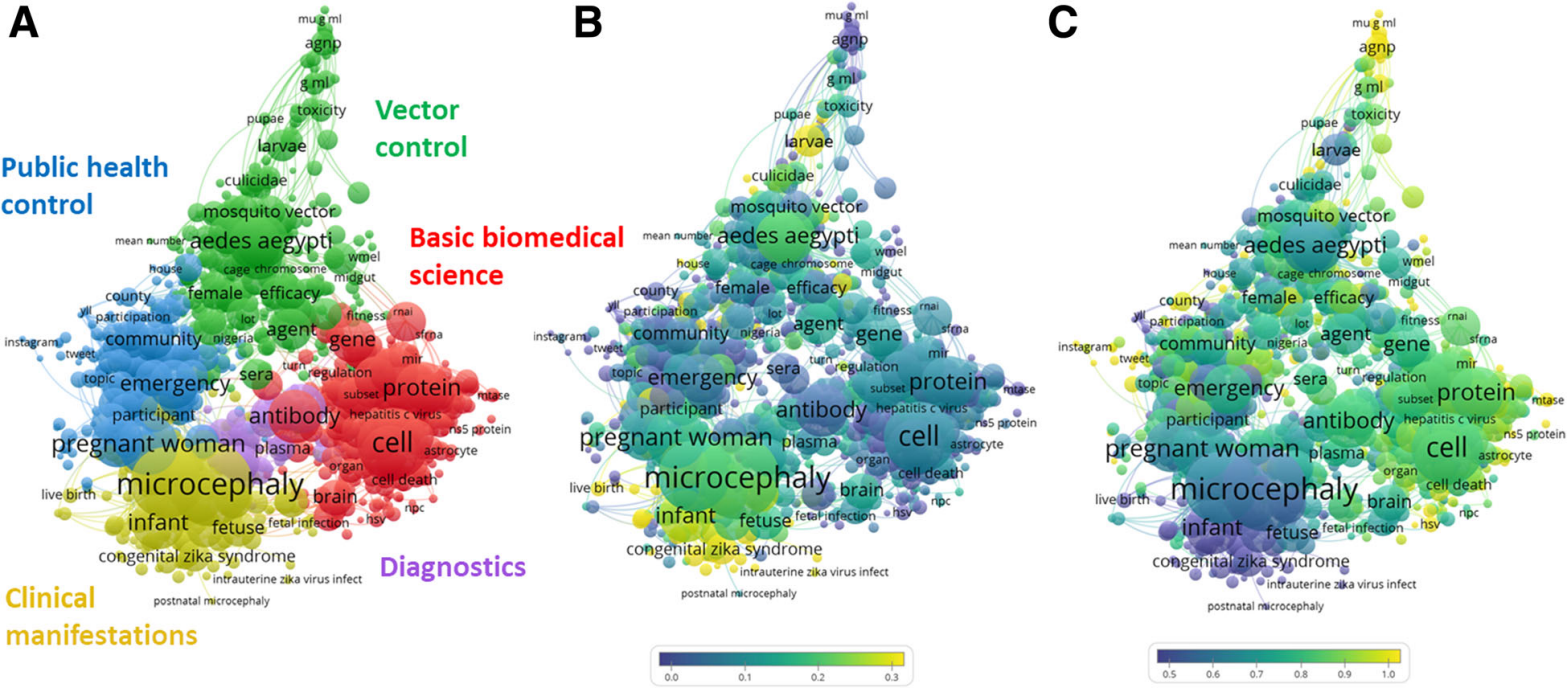
Term map of ZIKAV research. The map shows 1219 terms extracted from titles and abstracts of all ZIKAV publications (Global dataset). The closer two terms are located to each other, the stronger their relation. Each term is represented by a circle. The diameter and label size are proportional to their frequency in titles or abstracts. Each term displayed occurred in at least five publications. **a**) Colors indicate clusters of terms that have co-occurred more frequently in the dataset. **b** and **c**) Colors indicate the degree of occurrence of terms in publications authored by researchers based in LAC (**b**) or other countries (**c**), relative to the world average. Blue represents a low occurrence, green average, and red a high occurrence

To identify the research areas in which LAC countries mostly contributed, a term map of all publications in the Global dataset was built (Fig. 2a). Frequent terms appearing on titles and abstracts were automatically grouped and color-coded into five broad research areas/clusters. Each cluster represents a research area, identified by ZIKAV-related terms. Starting from the bottom left (yellow cluster) and moving clockwise, these were: i) clinical manifestations (microcephaly, pregnancy, congenital zika syndrome, malformation, abnormality, rash); ii) public health control (pregnant woman, emergency, zika virus disease, community, travel); iii) vector control (*Aedes aegypti*, species, agent, *Aedes albopictus*); iv) basic biomedical science (cell, mouse, protein, antibody, structure, gene); v) diagnostics (assay, urine, serum, saliva, sensitivity, PCR).

Research areas targeted in LAC publications (Fig. 2b) and research areas of other countries (Fig. 2c) are depicted as overlay visualizations of Fig. 2a. In these maps, blue represents a lower score, green an average, and red a higher score of occurrence of a term in publications in relation to the global average. LAC-based scientists have contributed more than the world average to research on “clinical manifestations”, particularly the ones related to effects on newborns. Researchers from other countries mostly contributed to “basic biomedical science” research, particularly cell biology and immunology. “Vector control” was of common interest for both LAC countries and the rest of the world, although their collaboration focused on ZIKAV clinical manifestations (data not shown).

### Brazil had a central role in the ZIKAV global research network

The Global dataset was used to build a global research network for ZIKAV. The network involved 156 countries, reflecting the solid international collaborative research efforts for disease control. The top three central countries, according to their betweenness centrality, were the USA, France, and Brazil (Table 2).

**Table 2:** Top five central countries in the ZIKAV global research network

Rank	Country	Betweenness centrality
1	USA	0.113
2	France	0.054
3	Brazil	0.043
4	United Kingdom	0.028
5	Switzerland	0.028

**Fig. 3:**
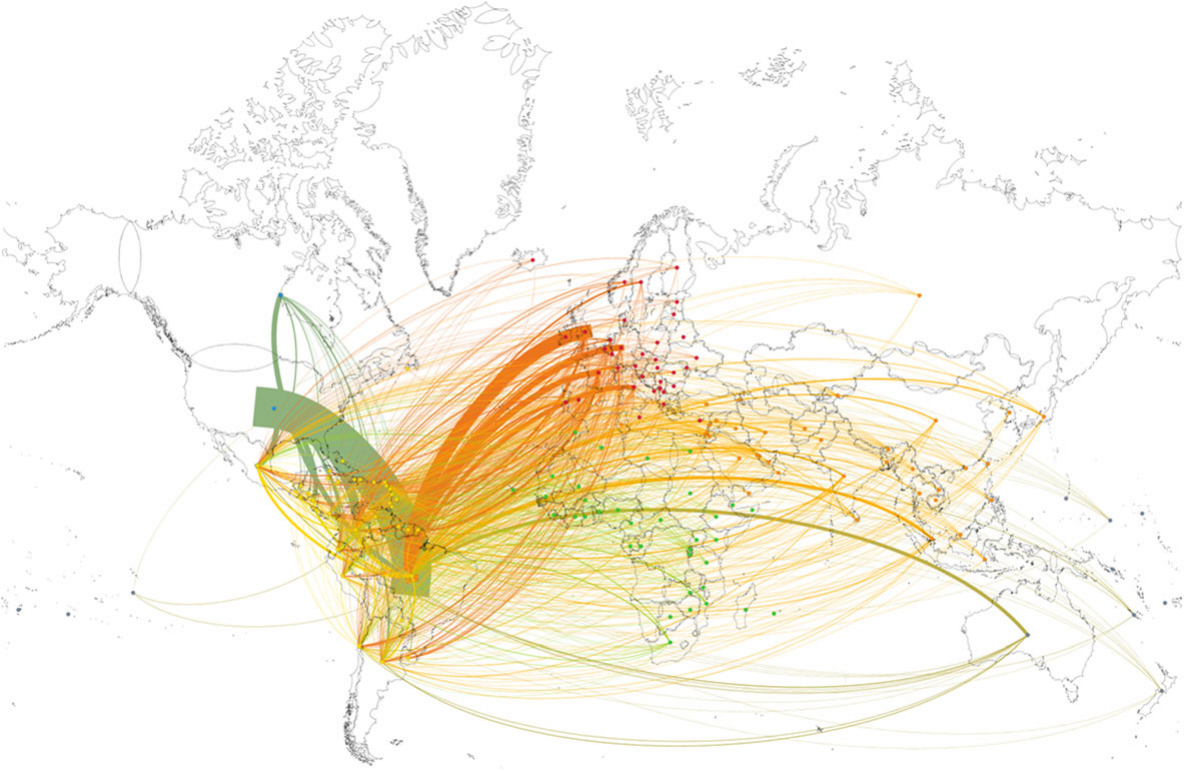
Global network of ZIKAV research involving LAC-based authors. Country links were mapped based on the authors’ affiliations. Each node represents one country, and two countries were considered connected if their researchers shared the authorship of a paper. The thickness of links indicates the frequency of collaboration between two nodes. For visualization purposes, only LAC countries collaborations, among themselves or with other countries, are shown. Nodes are color-coded according to the authors’ continent

Co-authorship network analyses showed that the USA was the most frequent collaborator of LAC countries (Fig. 3), co-authoring 19% (*n* = 210) of all LAC publications. Other frequent collaborating countries were the United Kingdom, Germany, and France, (*n* = 71, *n* = 34, and *n* = 30, respectively). Collaboration between LAC countries was not as frequent as expected. Brazilian researchers co-authored 17 publications with Colombian and 14 papers with Mexican authors.

### Brazil was the only LAC country filing patents on ZIKAV

A patent search was carried out to identify and assess LAC countries’ contributions to potential technological development towards ZIKAV. The search resulted in the retrieval of 417 patent families. Each of these contained one or more applications related to an invention, relative to, for instance, applications filed in more than one country. Detailed analysis indicated that only 274 families (65.7%) showed experimental evidence on ZIKAV and were selected for further analyses.

**Fig. 4:**
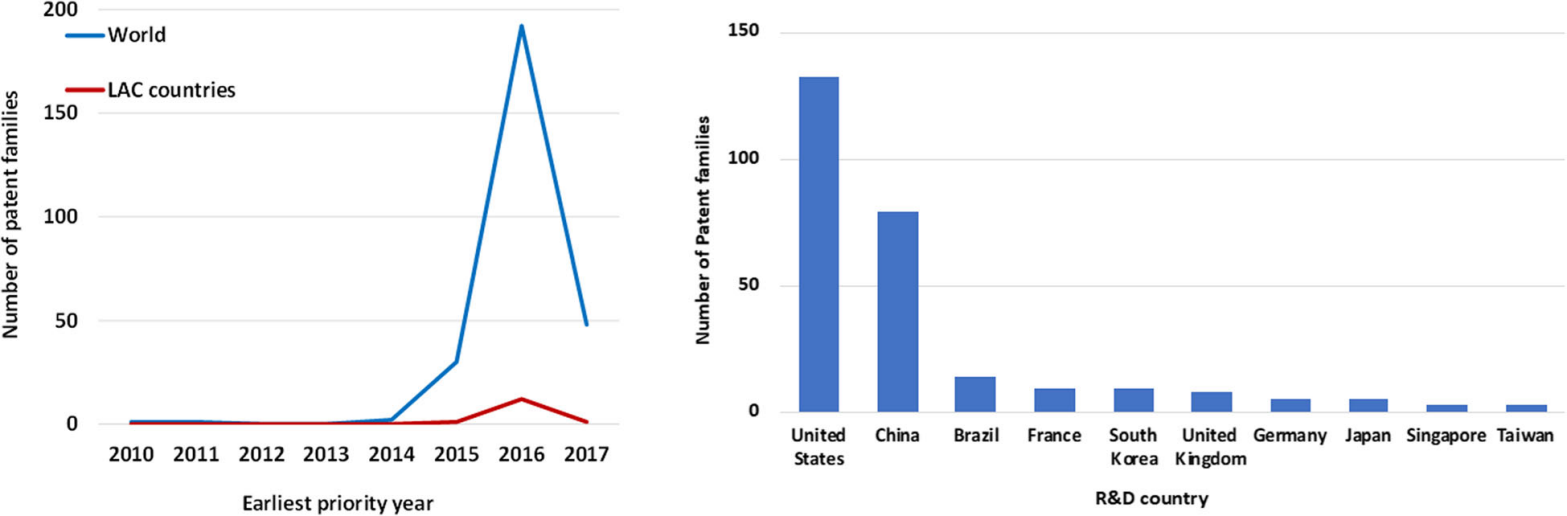
Patenting dynamics for ZIKAV and R&D origin (up to 2017). Left panel: number of patent families with experimental evidence on ZIKAV by earliest priority year (the year when the first patent in the family was filed). Right panel: number of patent families according to the R&D location. Only countries with three or more patent families are represented

To obtain a general portrait of the inventive activity related to ZIKAV, the number of patent families was plotted by their earliest priority year. In accordance with OECD’s recommendations, the earliest priority year (closest date to the invention) was selected as best indicator of inventive performance [19]. Filings started after 2010 with an increasing number from 2015. Approximately 87% of patent families had the earliest priority date from 2016 onwards (Fig. 4, left panel). LAC countries presented the same overall trend. As patents are usually published 18 months after the earliest priority claimed, it is possible that data for 2017 is not complete. The number of patent families first filed in 2017 should grow considerably when patents filed during and immediately after the 2015–2016 outbreak are published.

To infer where R&D activity took place, patent families were analyzed by inventor and assignee address. International collaboration was very limited, representing less than 3% of all cases. Seventy-five percent of all patent records were originated from assignees based in the USA and China and 5% from Brazilian-based assignees (Fig. 4, right panel). Brazil was the only LAC country filing patents.

The top countries of protection for ZIKAV patents were China, the USA, and Brazil. Canada, South Korea, and Australia follow suit, all with 10 or more live patents. Brazil, Mexico, Uruguay, Argentina, and Colombia were the only LAC countries where patent protection was currently sought (see Additional file 4).

### Most patent filings from Brazil came from independent inventors

**Fig. 5:**
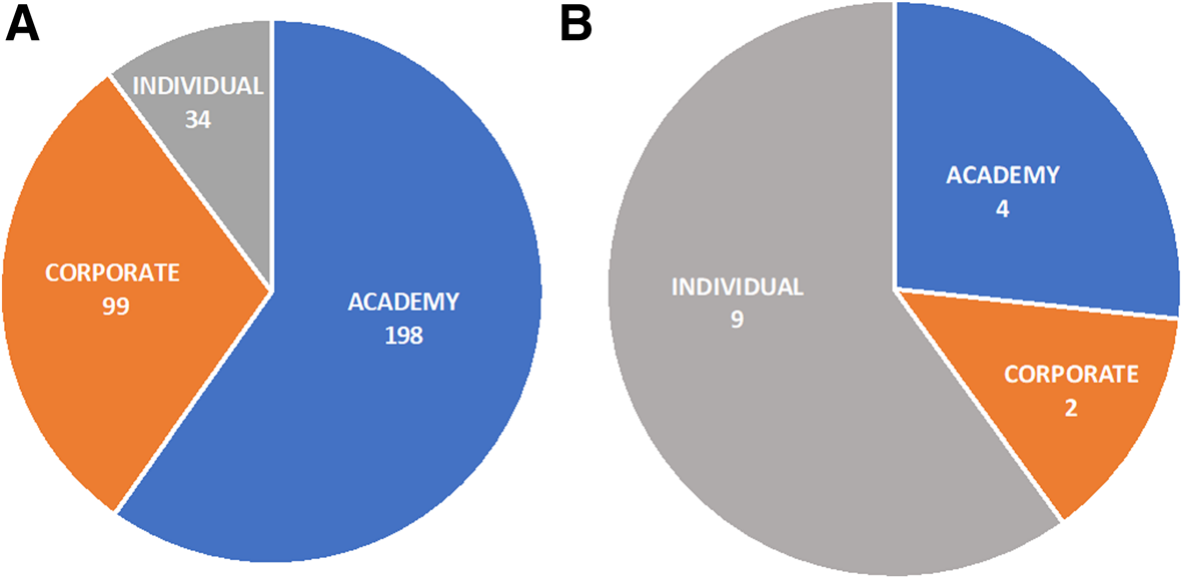
Distribution of ZIKAV patent families by assignee type. Assignees were classified and counted every time they were indicated as patent family assignee, even if they appeared in a previous patent family. **a**) The overall share of assignee types of the 274 patent families filed worldwide; **b**) Share of assignee types of the 14 patent families from Brazil

To provide information on the type of institutions behind inventive activity directed to ZIKAV, assignees were classified as “academy”, “corporate” or “independent inventor”. Overall, 60% of patent family assignees were from academic institutions, whereas 30% were corporate and 10% were independent inventors (Fig. 5a). A separate analysis of the 14 Brazilian patents indicated that 60% were from independent inventors, 27% from academic institutions and 13% from corporations (Fig. 5b).

Collaboration among institutions was limited. Globally, only 16% of patent families had more than one assignee. Most of these were academy-academy collaborations (59%) or academy-corporate (23%). The remaining were collaborations between independent inventors (11%), independent inventors and corporations (2%) or between companies (2%). In the Brazilian set of patents, only one collaboration between academic institutions and one between an independent inventor and a corporation were identified (data not shown).

### None of the leading patenting institutions was from Brazil

Assignees with four or more patent families are listed in Table 3. The best-ranked assignees were American (50%), Chinese (44%) and French (6%) institutions. Most of them (81%) were academic organizations. Brazilian assignees filed one patent family each and, therefore, did not appear in the rank.

**Table 3:** Top assignees of ZIKAV family patents. Only owners of four of more patent families are represented. Families were considered alive if they had at least one member still in force

Rank	Institution	Country	Number of records
1	Chinese Academy of Sciences	China	10
2	US Department of Health & Human Services	USA	10
3	Academy of Military Medical Sciences	China	7
4	Massachusetts Institute of Technology	USA	6
5	Capitalbio	China	5
6	Emory University	USA	5
7	Sun Yat Sen University	China	5
8	Tianjin Int Joint Acad of Biomedicine	USA	5
9	Centre National de la Recherche Scientifique (CNRS)	France	4
10	Harvard University	USA	4
11	Modernatx	USA	4
12	Sinovac	China	4
13	Third People S Hospital of Shenzhen	China	4
14	University of California	USA	4
15	University of Miami	USA	4
16	Southern Medical University	China	4

### Vector control was the main subject of Brazilian patents on ZIKAV

**Fig. 6:**
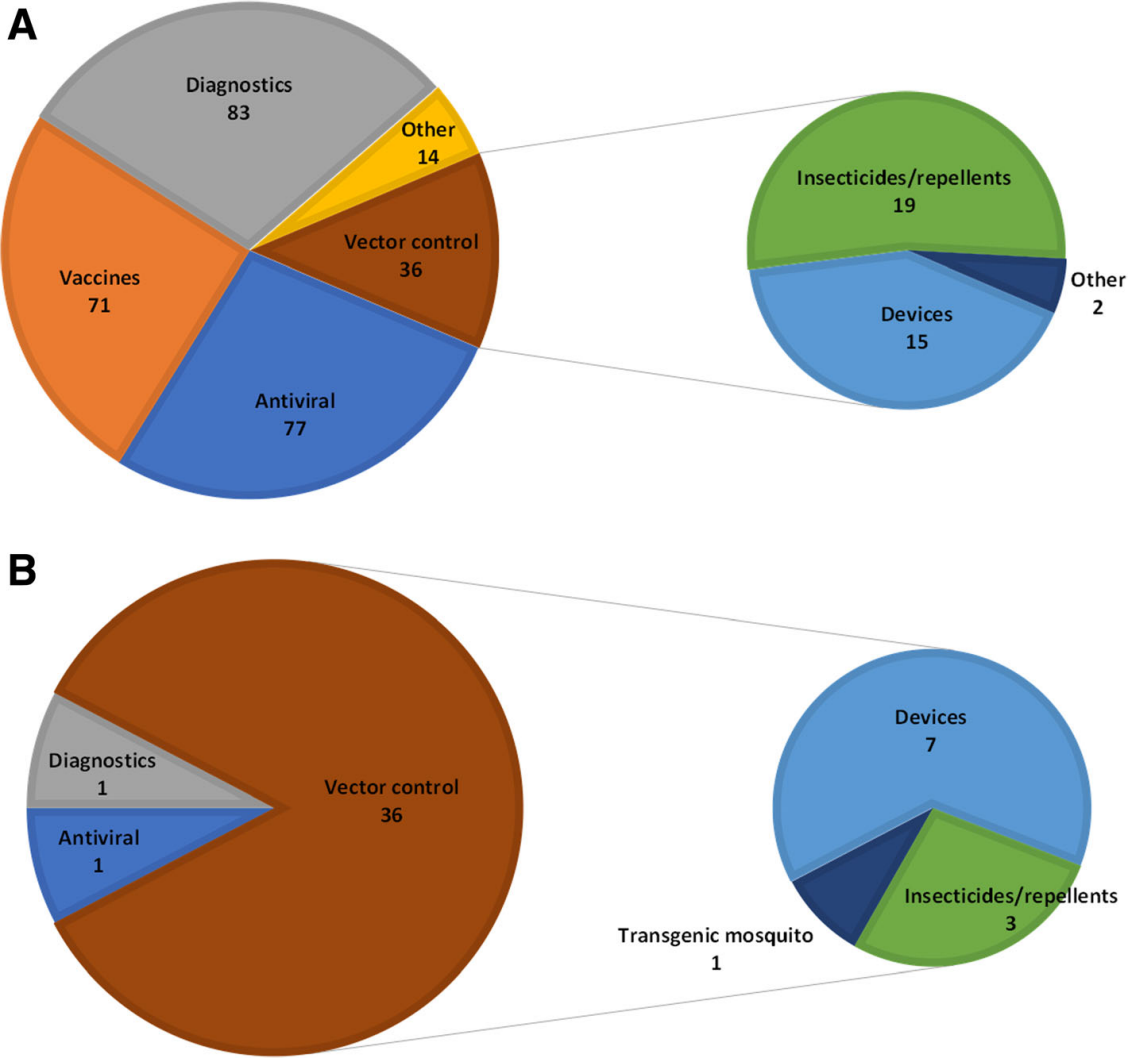
Classification of ZIKAV patent technologies. **a**) Overall classification of technologies of the 274 patent families filed worldwide; **b**) Classification of technologies of the 14 patent families from Brazil. “Vaccine” comprised both prophylactic and therapeutic vaccines

To characterize the inventions contemplated by ZIKAV patents, patent families were classified by technological area. Most patent families focused on diagnostic methods (30%), antiviral products (27%), and vaccine development (25%). A lower proportion of inventions (13%) focused on the disease vector. Further classification showed that most of these inventions were insecticides/repellents (53%) and devices for vector control (42%). The category “Other” (5%) included strategies for ZIKAV protein expression or antibody production, drug screening platforms, among others (Fig. 6a). Most patents from academic institutions were directed towards antivirals and diagnostic tests whereas corporations mostly focused on diagnostic tests and vaccines (data not shown).

Brazilian patents were mainly related to vector control (85%). From these, 64% encompassed devices for mosquito control, whereas 27% were on insecticides/repellents. One was a transgenic mosquito for vector control. The remaining inventions were a diagnostic test and a pharmaceutical for drug repurposing (Fig. 6b).

## Discussion

This study presents a comprehensive review of ZIKAV’s scientific knowledge production and potential technological development by combining the analyses of scientific publications, research networks, and patents. It gives an overview of the global S&T response to ZIKAV infection with special emphasis on LAC countries. The LAC region was the most affected area in the last outbreak and the region is expected to face the greatest public health impacts from ZIKAV disease. The analysis of regional contributions is relevant not only to inform future research and guide investments, but also to assess LAC countries’ potential role in tackling global health threats imposed by emerging diseases [13, 20, 21].

Our analyses confirmed the poor interest in ZIKAV R&D prior to 2015 and the striking S&T response after the LAC epidemics when severe clinical complications were attributed to the infection. WHO’s appeal for the opening of data and research information on ZIKAV was probably among the factors that promoted such a rapid reaction from the scientific community. It directly influenced the scientific communication by encouraging journals to “fast track” ZIKAV-related publications [22]. This increase in R&D activities was also bolstered by the funding made available by several international agencies [23, 24].

A reliable assessment of the LAC region’s scientific contributions in any given area is usually hindered by the use of international databases as unique sources of information [25, 26]. The impact of excluding regional databases is exacerbated by the trend of LAC authors to publish in regional and local journals due to languages barriers and costs of international journals [25]. Our review showed that 70% of all ZIKAV publications indexed in LAC regional databases were also available in the international databases. This indicated that an eventual bias due to low representation of regional articles was potentially reduced in this study. It seems that the global importance given to ZIKAV along with the high incidence of cases in LAC countries urged that knowledge produced regionally was made noticeable globally. Such global interest might also have influenced editorial decisions of international journals [9].

During an epidemic, the role played by countries in a knowledge-generating network is an important parameter for influencing response, decision-making, preparedness, and empowerment. Our analyses showed that scientific efforts from LAC countries, especially from Brazil, had a significant role in the generation of global knowledge on ZIKAV. Brazilian scientists were responsible for seminal work on outbreak characterization [27, 28] and documented the high incidence of microcephaly and the association of ZIKAV infection during pregnancy to newborn malformations [29–32].

The betweenness centrality analysis identified key countries acting as a “bridge” in the scientific community and suggested a prominent role for Brazil facilitating access to novel information and resources in the network. As a practical example of such role, when international scientists suggested that the 2016 Olympic Games should be put off Brazil because of the “Zika problem” [33], it was a report from Brazilian scientists that brought epidemiological evidence that the epidemic was in decay, and there was no increased risk for disease transmission [34].

In the patent analyses, Brazilian assignees were the single representatives of LAC countries. It should be kept in mind that, given the common delays in the publication of patent filings in the country, the number of patents may be underestimated. Although Brazil’s performance was less expressive than the USA and China, it still had an important position, ahead of patent leaders like South Korea, Germany, and Japan. Its leading position among LAC countries is in accordance with the results obtained in the assessment of scientific publications. Brazil’s small share of ZIKAV patents (5%) seems to be consistent with the publication’s profile, which focused mostly on the clinical manifestations of ZIKAV and vector control, areas that do not necessarily involve technological development. It has been argued that vector control is necessary as an immediate measure for epidemic control, but it should be undertaken in combination with other efforts such as the implementation of an R&D agenda and strengthening social programs and health systems [35].

Brazil also stands out as the main country of protection for the patent families filed in national offices, a natural reflection of the 2015–2016 outbreak. Assignees usually file patents in strategic countries for their inventions, such as the most promising markets, economically important regions, the assignee home country or the home country of potential licensors. Still, almost half of ZIKAV patents were filed via simplified patent systems (the Patent Cooperation Treaty system or the European Patent Office), which offer additional time for assignees to decide in which countries to protect their inventions. Given that most of these patents were filed from 2016 onwards, it is still unknown where protection will be sought for this share of patent families.

The markedly collaborative context in which ZIKAV scientific knowledge was generated was evidenced by the co-authorship network analyses. Indeed, several incentives to facilitate multidisciplinary research collaborations were set after the LAC epidemics, such as virtual databases [36]; the international research consortia ZikAction [37], ZikAlliance [38] and ZikaPlan [39]; the collaborative platforms LabKey Server [40] and the Global Research Collaboration for Infectious Disease Preparedness (GloPID-R) [41], among others. The multidisciplinary collaborative work was crucial to characterize the ZIKAV syndrome, in one of the most rapid and coordinated research responses against an emerging disease to date [42, 43]. In opposition, limited collaboration was evidenced in the patent analyses. However, given the lag time prior to the publication of patent applications, it is possible that the influence of research collaboration will only be observed in the next years.

The patent classification by assignee type evidenced a major role of the academy and academic assignees, as expected from the active response from the scientific community to the 2015–2016 outbreak. Diagnostics was one of the major areas targeted in patent documents, also present in scientific publications.

In Brazil, individuals without affiliation accounted for 60% of the total patent families, whereas the academy was responsible for only 27% of the filings. This may be explained by the focus on vector control, involving the development of low-tech traps and nets, which could possibly be developed without institutional support. According to the WHO, innovative vector control tools that reduce the mosquito population are important technological contributions, amongst the most viable R&D options to help fight the spread of ZIKAV in the immediate future [44].

This study focused on publications and patents, and it may not include all contributions of LAC countries towards ZIKAV prevention, treatment, and control. However, the S&T indicators described herein are considered valuable tools in policy studies as they objectively assess the diffusion and impact of research, and disclose the geographic origin of the contribution. The use of co-authorship data as an indicator of scientific collaboration has limitations, but, in most cases, it indicates active cooperation in addition to the simple exchange of material or information. We recognize that reviewing only patents with experimental evidence could have resulted in an underestimation of the number of ZIKAV patents filed. We believe that the inclusion of all patent documents regardless of experimental evidence would have produced disputed results.

## Conclusions

We reviewed scientific publications, patent records, and co-authorship networks to provide a broad scenario of LAC engagement in research and technological development towards ZIKAV prevention, treatment, and control. The information presented herein has value in informing the global health community and policymakers that (i) LAC countries had a key role in generating and disseminating scientific knowledge on ZIKAV, suggesting a strong research capacity; (ii) LAC countries have expressively contributed to research on ZIKAV clinical manifestations, especially the ones related to effects on newborns; (iii) the Brazilian scientific community was potentially very effective in knowledge sharing and diffusion in the ZIKAV research network, indicating a solid capacity to incentivize and coordinate future LAC collaboration; (iv) Brazil was the single LAC country filing patents, mostly represented by independent inventors and low-tech patents, indicating the need to invest in more technologically advanced areas.

Finally, the high population concentration in major LAC cities and the tropical climate suggest that the region remains vulnerable to ZIKAV and other vector-borne diseases. The need for a continued collaborative and interdisciplinary work, as well as long-term support to strengthen local leadership, is critical to improving LAC capability to prepare for, control and prevent future outbreaks.

## Additional files

Additional file 1: ZIKAV-related publications up to December 2017, retrieved from WoS, PubMed, and Scopus. (CSV 1513 kb)

Additional file 2: ZIKAV-related patents with earliest priority date up to 12/31/2017 (XLSX 1728 kb)

Additional file 3: Overlap between databases. Left: overlap between PubMed, Scopus, and WoS. Right: Overlap between SciELO, LILACS and the three international databases. (TIF 3429 kb)

Additional file 4: Markets of interest for ZIKAV. The number of live individual patent counts (FullPat) is shown by country of filing, indicating where protection was sought. Only countries with 2 or more live patents are represented in the main graph. LAC countries are represented in the side graph. Country codes: Argentina (AR), Australia (AU), Belgium (BE), Brazil (BR), Canada (CA), China (CN), Colombia (CO), Europe (EP), France (FR), India (IN), Israel (IL), Japan (JP), Mexico (MX), PCT countries (WO), Philippines (PH), Russia (RU), Singapore (SG), South Korea (KR), Taiwan (TW), United Kingdom (GB), United States (US), and Uruguay (UY). (TIF 2858 kb)

## Abbreviations

LAC: Latin America and the Caribbean
LILACS: Literature in the Health Sciences in Latin America and the Caribbean
OECD: Organisation for Economic Co-operation and Development
R&D: Research and development
S&T: Science and technology
SciELO: Scientific Electronic Library Online
WHO: World Health Organization
ZIKAV: Zika virus
