# Supplementary figures and images for: Scientific and technological contributions of Latin America and Caribbean countries to the Zika virus outbreak
Source: BMC Public Health. 2019 May 9;19:530. doi: 10.1186/s12889-019-6842-x (PMC6507135; doi:10.1186/s12889-019-6842-x)

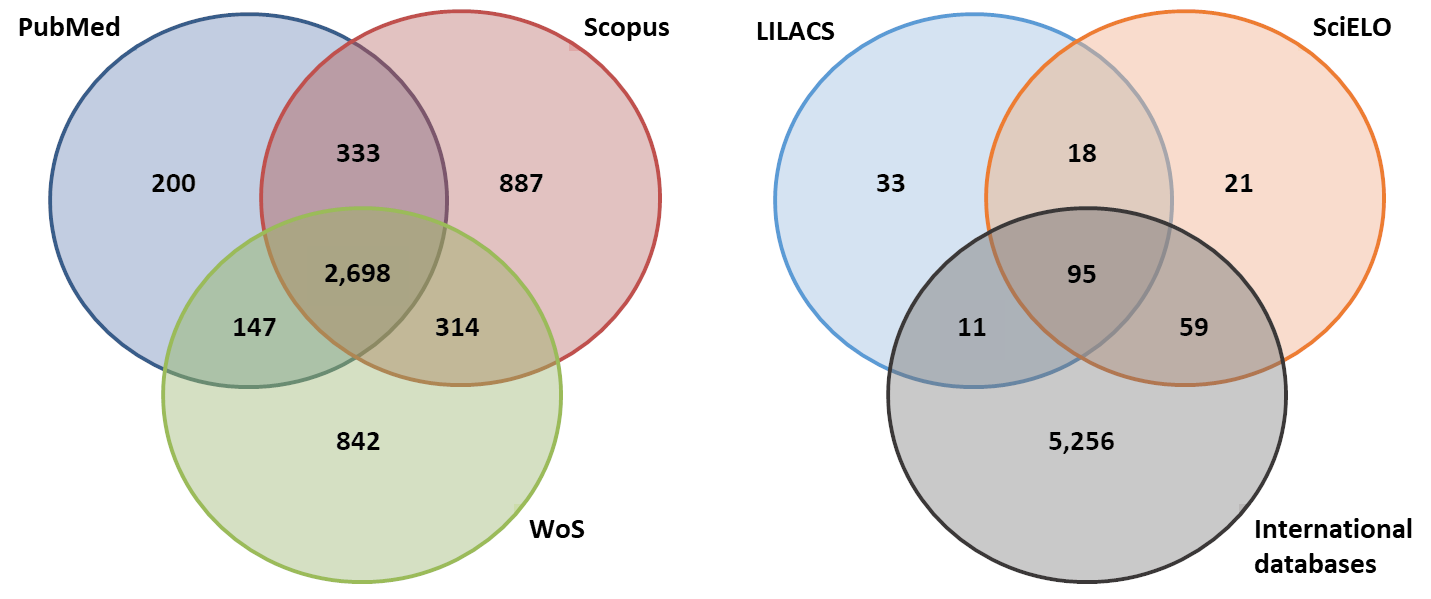

Supplement: Supplementary file 3 — Overlap between databases. Left: overlap between PubMed, Scopus, and WoS. Right: Overlap between SciELO, LILACS and the three international databases. (TIF 3429 kb) [file 12889_2019_6842_MOESM3_ESM.tif]

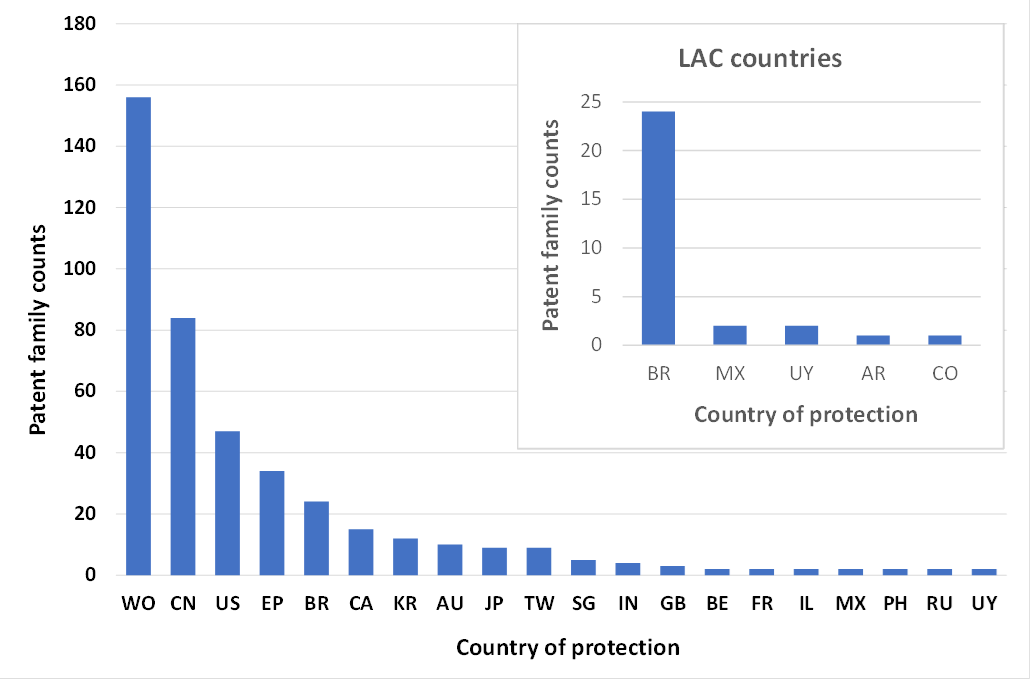

Supplement: Supplementary file 4 — Markets of interest for ZIKAV. The number of live individual patent counts (FullPat) is shown by country of filing, indicating where protection was sought. Only countries with 2 or more live patents are represented in the main graph. LAC countries are represented in the side graph. Country codes: Argentina (AR), Australia (AU), Belgium (BE), Brazil (BR), Canada (CA), China (CN), Colombia (CO), Europe (EP), France (FR), India (IN), Israel (IL), Japan (JP), Mexico (MX), PCT countries (WO), Philippines (PH), Russia (RU), Singapore (SG), South Korea (KR), Taiwan (TW), United Kingdom (GB), United States (US), and Uruguay (UY). (TIF 2858 kb) [file 12889_2019_6842_MOESM4_ESM.tif]
